# Chondroitin and glucosamine sulfate in combination decrease the pro-resorptive properties of human osteoarthritis subchondral bone osteoblasts: a basic science study

**DOI:** 10.1186/ar2325

**Published:** 2007-11-09

**Authors:** Steeve Kwan Tat, Jean-Pierre Pelletier, Josep Vergés, Daniel Lajeunesse, Eulàlia Montell, Hassan Fahmi, Martin Lavigne, Johanne Martel-Pelletier

**Affiliations:** 1 Osteoarthritis Research Unit, University of Montreal Hospital Centre, Notre-Dame Hospital, 1560 rue Sherbrooke Est, Montreal, Quebec H2L 4M1, Canada; 2 Scientific Medical Department, Bioiberica, S.A., Pza Francesc Macià 7, Barcelona 08029, Spain; 3 Department of Orthopaedics, Maisonneuve-Rosemont Hospital, 5345 boulevard l'Assomption, Montreal, Quebec H1T 4B3, Canada

## Abstract

Early in the pathological process of osteoarthritis (OA), subchondral bone remodelling, which is related to altered osteoblast metabolism, takes place. In the present study, we explored in human OA subchondral bone whether chondroitin sulfate (CS), glucosamine sulfate (GS), or both together affect the major bone biomarkers, osteoprotegerin (OPG), receptor activator of nuclear factor-kappa B ligand (RANKL), and the pro-resorptive activity of OA osteoblasts. The effect of CS (200 μg/mL), GS (50 and 200 μg/mL), or both together on human OA subchondral bone osteoblasts, in the presence or absence of 1,25(OH)_2_D_3 _(vitamin D_3_) (50 nM), was determined on the bone biomarkers alkaline phosphatase and osteocalcin, on the expression (mRNA) and production (enzyme-linked immunosorbent assay) of bone remodelling factors OPG and RANKL, and on the pro-resorptive activity of these cells. For the latter experiments, human OA osteoblasts were incubated with differentiated peripheral blood mononuclear cells on a sub-micron synthetic calcium phosphate thin film. Data showed that CS and GS affected neither basal nor vitamin D_3_-induced alkaline phosphatase or osteocalcin release. Interestingly, *OPG *expression and production under basal conditions or vitamin D_3 _treatment were upregulated by CS and by both CS and GS incubated together. Under basal conditions, *RANKL *expression was significantly reduced by CS and by both drugs incubated together. Under vitamin D_3_, these drugs also showed a decrease in *RANKL *level, which, however, did not reach statistical significance. Importantly, under basal conditions, CS and both compounds combined significantly upregulated the expression ratio of *OPG/RANKL*. Vitamin D_3 _decreased this ratio, and GS further decreased it. Both drugs reduced the resorption activity, and statistical significance was reached for GS and when CS and GS were incubated together. Our data indicate that CS and GS do not overly affect cell integrity or bone biomarkers. Yet CS and both compounds together increase the expression ratio of *OPG/RANKL*, suggesting a positive effect on OA subchondral bone structural changes. This was confirmed by the decreased resorptive activity for the combination of CS and GS. These data are of major significance and may help to explain how these two drugs exert a positive effect on OA pathophysiology.

## Introduction

Osteoarthritis (OA) is one of the most common joint disorders, affecting approximately 65% of individuals over 60 years of age, many of whom suffer from pain and functional disability, and resulting in a significant social and economic burden. Despite the high prevalence of OA, its precise etiopathogenesis is not yet completely understood, although significant progress has been made in the last few decades.

OA is considered a complex illness in which tissues of the joint, including cartilage, synovial membrane, and subchondral bone, play significant roles [1]. Even though articular cartilage destruction is a major characteristic of OA, we still do not completely understand what initiates its degradation and loss. Synovial membrane inflammation is believed to play an important role in the progression of joint tissue lesions; however, there is a general consensus that synovial inflammation in OA is not the primary cause of the disease but rather a secondary phenomenon related to multiple factors, including cartilage matrix degradation. Moreover, studies have also demonstrated that, in OA, the subchondral bone is not an innocent bystander but is the site of several dynamic morphological changes that appear to be part of the disease process [2]. These changes are associated with a number of local abnormal biochemical pathways related to the altered osteoblast metabolism.

Some compounds have been shown to have a slow-acting symptomatic effect in OA and are termed SYSADOA [3]. Among this group of pharmacological substances are chondroitin sulfate (CS and glucosamine sulfate (GS). Several strategies have been investigated for the symptomatic and structural management of OA using these two drugs. There is compelling evidence of the potential for inhibiting the structural progression of OA with CS and GS in patients with OA of the knees and hands [4-6] Moreover, the recent Glucosamine/Chondroitin Arthritis Intervention Trial suggests, following exploratory analyses, that the combination of the two drugs was effective on symptoms in OA patients having moderate to severe knee pain [7].

Glucosamine is an aminosaccharide that acts as a preferred substrate for the biosynthesis of glycosaminoglycan (GAG chains and, subsequently, for the production of aggrecan and other proteoglycans. CS is a major component of the extracellular matrix of many connective tissues, including cartilage, bone, skin, ligaments, and tendons. It is a sulfated GAG composed of a long unbranched polysaccharide chain with a repeating disaccharide structure of *N*-acetylgalactosamine and glucuronic acid. Most of the *N*-acetylgalactosamine residues are sulfated, particularly in the 4- or 6-position, making it a strongly charged polyanion. A number of small leucine-rich proteoglycans, especially decorin and biglycan (which contain high levels of CS chains), are present in the bone extracellular matrix compartment [8]. In OA articular tissues, changes in the structure of CS have been reported, with the appearance of longer chains [9]. In *in vitro *studies, both GS and CS have demonstrated the ability to diminish pro-inflammatory factors, to modify the cellular death process, and to improve the anabolism/catabolism balance of extracellular cartilage matrix. In addition, CS has proven to have a positive effect on OA synovial membrane. However, the exact mechanisms of action underlying their beneficial effects remain poorly understood, and their action on the factors involved in subchondral bone remodelling has never been investigated.

Although sclerosis of the subchondral bone is seen at a late stage of the OA process, several *in vitro *and *in vivo *[10] reports have indicated that subchondral bone remodelling involving bone resorption occurs early in the disease. Osteoblasts from human OA subchondral bone have been shown to produce an excess of many biochemical factors favoring the maturation/activation of osteoclasts and/or resorption of bone matrix. Abnormal levels of two major factors that play a major role in bone resorption, osteoprotegerin (OPG) and the receptor activator of nuclear factor-kappa B ligand (RANKL), have been found in human OA subchondral bone osteoblasts [11]. Both factors are synthesized by osteoblasts, and RANKL, a member of the tumour necrosis factor superfamily, is an essential cytokine for osteoclast differentiation and bone loss. On the other hand, OPG is considered a decoy receptor that blocks the binding of RANKL to the RANK receptor, located on osteoclast precursors, thereby inhibiting the terminal stage of osteoclastic differentiation and suppressing its activation as well as inducing the apoptosis of mature osteoclasts. Thus, OPG, by preventing osteoclastogenesis, inhibits bone resorption.

Recent data showed that human OA subchondral bone osteoblasts could be discriminated into two groups according to low (L) or high (H) OA osteoblasts based on the level of prostaglandin E_2 _(PGE_2 _production [12,13].(Interestingly, we further showed that L-OA osteoblasts promote osteoclast differentiation and formation and an increase in *RANKL *levels leading to a decreased *OPG/RANKL *expression ratio in favor of bone destruction [11]. However, the H-OA osteoblasts appear to be under the influence of factors favoring bone deposition [11,14].

In the present study, we explored in human subchondral bone whether CS and GS or both together affect certain bone biomarkers, OPG and RANKL levels, and pro-resorptive activity. Data showed that neither CS nor GS overly affects cell integrity or osteoblast phenotypic cell markers. However, CS and both CS and GS together significantly increased the expression ratio of *OPG/RANKL*, and GS and both CS and GS significantly decreased the OA osteoblast pro-resorptive activity. This suggests that these drugs could have a positive effect on OA subchondral bone structural changes, explaining the *in vivo *beneficial effect of CS and GS alone or combined.

## Materials and methods

### Specimen selection

Human OA specimens were obtained from the femoral condyles of patients undergoing total knee arthroplasty (mean age ± standard deviation: 74 ± 9 years). All patients were evaluated as having OA according to the American College of Rheumatology clinical criteria [15]. At the time of surgery, the patients had symptomatic disease requiring medical treatment in the form of acetaminophen, nonsteroidal anti-inflammatory drugs, or selective cyclooxygenase-2 inhibitors. None had received intra-articular steroid injections within 3 months prior to surgery, and none had received medication that would interfere with bone metabolism. The institutional ethics committee board of Notre-Dame Hospital (Montreal, QC, Canada) approved the use of the human articular tissues.

### Subchondral bone osteoblast culture

The subchondral bone osteoblast culture was prepared as previously described [16,17] The overlying cartilage was removed and the trabecular bone tissue was dissected from the subchondral bone plate. All manipulations were performed under a magnifying microscope to ensure complete removal of cartilage and trabecular bone. Briefly, bone samples were cut into small pieces prior to sequential digestion in the presence of collagenase type I in BGJb medium (both from Sigma-Aldrich, Oakville, ON, Canada) without serum at 37°C in a humidified atmosphere of 5% CO_2_/95% air. After a 4-hour incubation period, the bone pieces were cultured in BGJb medium containing 20% heat-inactivated fetal calf serum (FCS) (Gibco-BRL, now part of Invitrogen Corporation, Carlsbad, CA, USA) and an antibiotic mixture (100 units/mL penicillin base and 100 μg/mL streptomycin base; Invitrogen Corporation) at 37°C in the humidified atmosphere. This medium was replaced every 2 days until cells were observed in the Petri dishes. At this point, the culture medium was replaced with fresh medium containing 10% FCS until confluence. Osteoblasts were passaged once and grown until confluence (about 5 days) in Dulbecco's modified Eagle's medium (DMEM) containing 10% FCS. Of note, osteoblasts from human subchondral bone, as prepared, have been shown to be mature differentiated cells since they express the bone-specific markers, including alkaline phosphatase and osteocalcin [12,13,16-21].

Cells were seeded at high density (200,000 cells/12 wells per plate) and cultured to confluence. They were treated with CS, GS, or both together in the absence or presence of 1,25(OH)_2_D_3 _(vitamin D_3_). The concentrations used were 200 μg/mL for CS (CS Bio-Active; Bioiberica, S.A., Barcelona, Spain), 50 and 200 μg/mL for GS (Bioiberica, S.A.), 200 μg/mL for CS and GS when used together, and 50 nM for vitamin D_3 _(Sigma-Aldrich). The effect of the factors was assessed by pre-incubating confluent cells in DMEM (Invitrogen Corporation)/0.5% FCS for 24 hours followed by an incubation of 18 hours (for mRNA determination) and 48 hours (for protein determination) with the factors under study. Preliminary experiments in which time course (0, 2, 8, 18, and 36 hours) was performed for the expression of *OPG *and *RANKL *under CS and GS treatment revealed a maximum effect on both *OPG *and *RANKL *at 18 hours.

### RNA extraction, reverse transcription, and real-time polymerase chain reaction

Total cellular RNA from human osteoblasts was extracted with the TRIzol™ reagent (Invitrogen Corporation) according to the manufacturer's specifications and treated with the DNA-free™ DNase Treatment and Removal kit (Ambion, Inc., Austin, TX, USA) to ensure complete removal of chromosomal DNA. The RNA was quantitated using the RiboGreen RNA quantitation kit (Molecular Probes Inc., now part of Invitrogen Corporation). The reverse transcription (RT) reactions were primed with random hexamers as described previously [22]. Real-time quantitation of mRNA was performed as previously described [22] in the GeneAmp 5700 Sequence Detection System (Applied Biosystems, Foster City, CA, USA) with the 2× Quantitect SYBR Green PCR [polymerase chain reaction] Master Mix (Qiagen, Mississauga, ON, Canada) used according to the manufacturer's specifications. In brief, 45 ng of the cDNA obtained from the RT reactions was amplified in a total volume of 50 μL consisting of 1× Master mix, uracil-*N*-glycosylase (Epicentre Biotechnologies, Madison, WI, USA) 0.5 units, and the gene-specific primers, which were added at a final concentration of 200 nM. The primer sequences were 5'-GTTTACTTTGGTGCCAGG (antisense) and 5'-GCTTGAAACATAGGAGCTG (sense) (*OPG*), 5'-GGGTATGAGAACTTGGGATT (antisense) and 5'-CACTATTAATGCCACCGAC (sense) (*RANKL*), and 5'-CAGAACATCATCCCTGCCTCT (antisense) and 5'-GCTTGACAAAGTGGTCGTTGAG (sense) (glyceraldehyde-3-phosphate dehydrogenase [*GAPDH*]). The primer efficiencies for the test genes were the same as for the *GAPDH *gene. The standard curves were generated with the same plasmids as the target sequences. The data were collected and processed with GeneAmp 5700 SDS software and given as a threshold cycle (C_T_) corresponding to the PCR cycle at which an increase in reporter fluorescence above a baseline signal can first be detected. The C_T _was then converted to number of molecules, and the values for each sample were calculated as the ratio of the number of molecules of the target gene to the number of molecules of *GAPDH*. Data are expressed as arbitrary unit over the control, which was given 1 as unit.

### Protein determinations

As previously described in the literature [12,13,16,17,23,24], the activity of alkaline phosphatase, osteocalcin, and PGE_2 _was determined after a 48-hour incubation. The alkaline phosphatase was determined in cell lysate, and the levels of osteocalcin, OPG, and PGE_2 _in the culture media. Alkaline phosphatase activity was determined by substrate hydrolysis using p-nitrophenylphosphate [16], osteocalcin using an enzyme immunoassay (EIA) (Biomedical Technologies Inc., Stoughton, MA, USA) with a sensitivity of 0.5 ng/mL, OPG by an enzyme-linked immunosorbent assay (MediCorp Inc., Montreal, QC, Canada) with a sensitivity of 2.8 pg/mL, total soluble RANKL by an EIA (ALPCO Diagnostics, Salem, NH, USA) with a sensitivity of 30 pg/mL, and PGE_2 _by an EIA (Cayman Chemical Company, Ann Arbor, MI, USA) with a sensitivity of 7.8 pg/mL. The protein concentration was determined using the bicinchoninic acid method (Pierce, Rockford, IL, USA). All determinations were performed in duplicate for each cell culture.

### Resorption activity determination

The BD BioCoat Osteologic Bone Cell Culture System (BD Biosciences, Oakville, ON, Canada) was used. Briefly, this methodology consists of sub-micron synthetic calcium phosphate thin films coated onto culture vessels. In brief, human peripheral blood mononuclear cells (PBMCs) (100,000 cells/well) were inoculated onto the wells with culture media containing DMEM/10% FCS, antibiotics, and 25 ng/mL macrophage colony-stimulating factor (M-CSF) and incubated for 3 days at 37°C in a humidified atmosphere in order to induce pre-osteoclastic differentiation [25]. Human OA subchondral bone osteoblasts (10,000 cells/well) were then inoculated with the differentiated PBMCs (pre-osteoclast) and incubated for another 3 days in fresh culture medium. At the end of this period, culture medium was eliminated and cells were incubated in DMEM containing M-CSF, 10% FCS, and antibiotics for 3 weeks with factors under testing. Media were changed every 3 days. Incubation was carried out at 37°C. At the end of the incubation period, cells were bleached (6% NaOCl, 5.2% NaCl) and extensively washed in sterilized water. Von Kossa stain was used for contrast as described by BD Biosciences. In brief, the films were stained with fresh silver nitrate (5%) for 10 minutes and washed extensively, and stains were developed with fresh sodium carbonate (5%) in formalin (25%) for approximately 30 seconds. The films were washed again and fixed with sodium thiosulfate (5%) for 2 minutes and washed. The quantitation was performed with the use of a light microscope with the Bioquant software (Bioquant Osteo II, v 8.00.20; BIOQUANT Image Analysis Corporation, Nashville, TN, USA). Results are represented as the mean resorbed surface per total surface.

To rule out that CS or GS directly affects PBMC differentiation in osteoclasts, experiments were performed as above with the PBMCs only, and also with osteoblasts, and the number of differentiated osteoclasts was measured with the tartrate-resistant acid phosphatase (TRAP) using the Bioquant software. At the end of the incubation period, the cells were fixed and stained for TRAP according to the manufacturer's recommendation (Sigma-Aldrich).

### Statistical analysis

Data are expressed as the mean ± standard error of the mean. Statistical significance was assessed by a two-tailed paired Student *t *test. *P *values of less than 0.05 were considered significant.

## Results

### Human osteoarthritis subchondral bone osteoblast classification

We previously showed that patients with OA can be discriminated into two groups classified according to L- or H-OA osteoblasts based on the level of PGE_2 _production [12,13] and that L-OA osteoblasts (PGE_2 _levels of less than 2,000 pg/mg protein) were suggested to favor pro-resorptive activity whereas the H-OA osteoblasts favor bone deposition [11,14] To investigate the effects of the compounds on (among other things) the OPG, RANKL, and pro-resorptive activity levels, we chose to perform this study with the L-OA osteoblast specimens. In this study, the OA subchondral bone osteoblasts used had a PGE_2 _level of 563.4 ± 115.0 pg/mg protein.

### Osteoblast biomarkers

**Figure 1:**
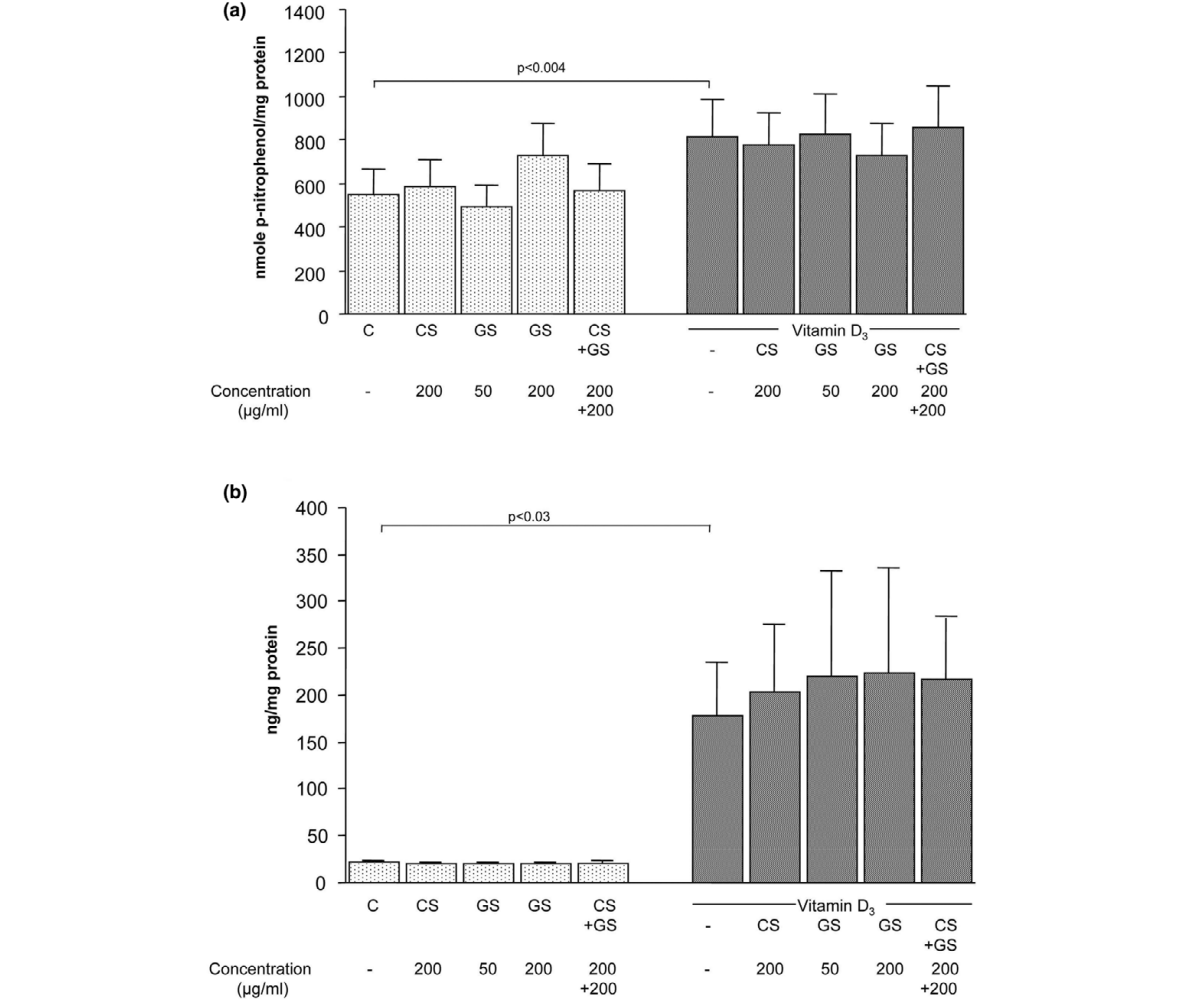
Levels of alkaline phosphatase and osteocalcin in human osteoarthritis subchondral bone osteoblasts. Alkaline phosphatase activity **(a) **and osteocalcin level **(b) **were determined after treatment with chondroitin sulfate (CS) (200 μg/mL), glucosamine sulfate (GS) (50 or 200 μg/mL), or both (200 μg/mL each) in the absence or presence of vitamin D_3 _at 50 nM. Alkaline phosphatase activity **(a) **was determined in the cell lysate by substrate hydrolysis using p-nitrophenylphosphate, whereas osteocalcin level **(b) **was determined in the culture media by using a specific enzyme immunoassay. Data are from eight independent experiments. Statistical significance was assessed by paired Student *t *test. *P *value indicates the statistical difference between control (C, basal conditions) and vitamin D_3_-treated specimens.

Previous studies with human OA subchondral osteoblasts have shown that these cells have abnormal bone biomarker levels [12,13,16,17](In this study, we first looked at two such biomarkers, namely alkaline phosphatase and osteocalcin. Data showed (Figure 1a,b) that alkaline phosphatase activity and osteocalcin responded to vitamin D_3_, as is expected from human subchondral bone osteoblasts, with approximately 1.5- and 8-fold increases for alkaline phosphatase and osteocalcin, respectively, over basal values. Neither alkaline phosphatase nor osteocalcin was truly affected by CS or GS alone or together; this is true for both basal conditions and hormonal stimulation. There was a tendency for all treated specimens to show higher levels of vitamin D_3_-induced osteocalcin release, yet this failed to reach statistical significance (Figure 1b).

### Osteoprotegerin and RANKL expression and synthesis

**Figure 2:**
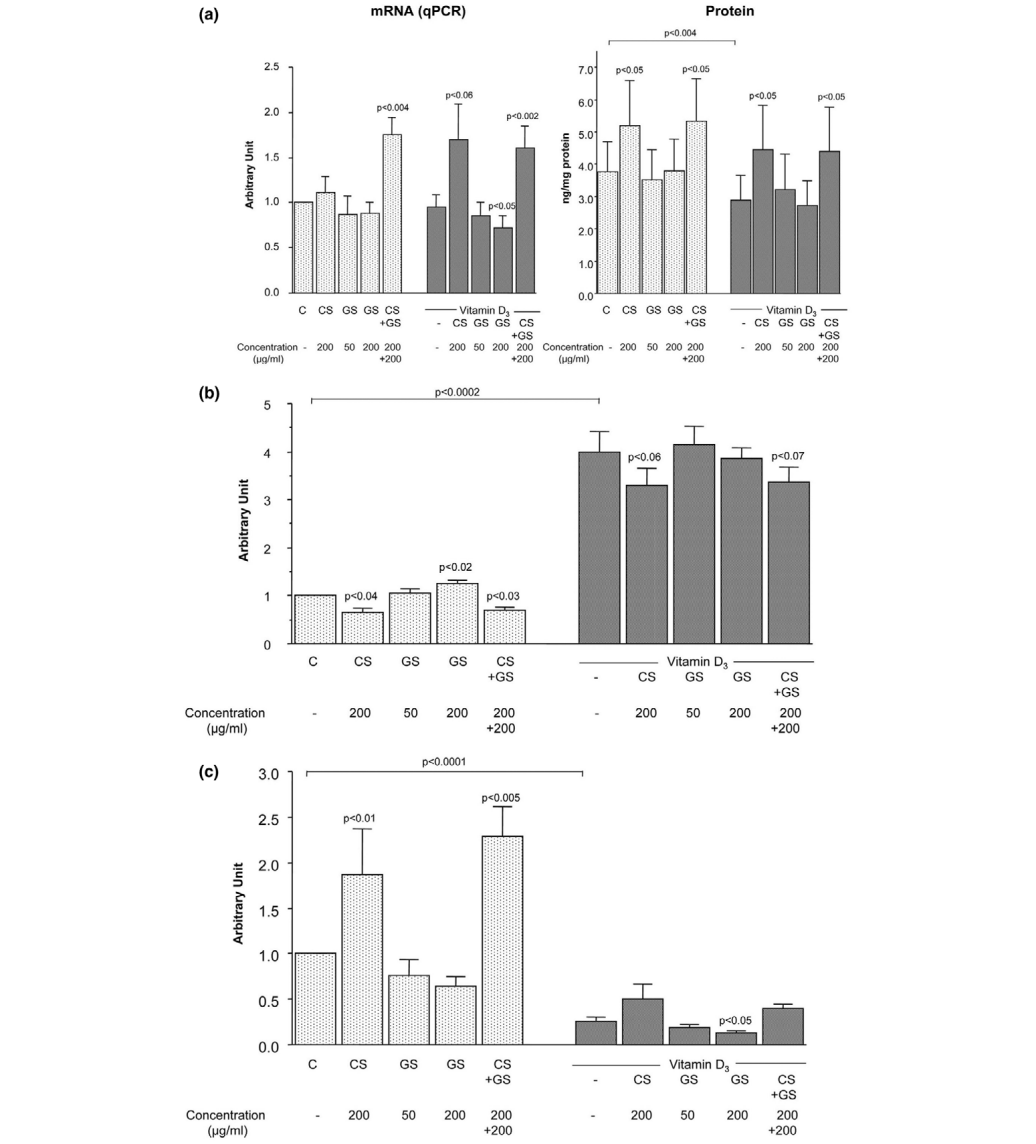
Levels of osteoprotegerin (*OPG*), receptor activator of nuclear factor-kappa B ligand (*RANKL*), and *OPG/RANKL *ratio in human osteoarthritis subchondral bone osteoblasts. Expression and production of *OPG ***(a)**, expression of *RANKL ***(b)**, and expression ratio of *OPG/RANK*L **(c) **of cells incubated in the absence or presence of chondroitin sulfate (CS) (200 μg/mL), glucosamine sulfate (GS) (50 or 200 μg/mL), or both (200 μg/mL each) in the absence or presence of vitamin D_3 _at 50 nM. Total RNA was extracted and processed for quantitative polymerase chain reaction (qPCR), and the data are expressed as the mean ± standard error of the mean of arbitrary unit. The release of *OPG *was determined in the culture medium by a specific enzyme-linked immunosorbent assay. Data are from eight independent experiments. Statistical significance was assessed by paired Student *t *test versus autologous control. Underlined *p *value indicates the statistical difference between control (C, basal conditions) and vitamin D_3_-treated specimens.

*OPG *expression (Figure 2a) was not altered by treatment with vitamin D_3_. Under basal conditions, *OPG *expression was found to be significantly increased when CS and GS were incubated together. CS showed an increased level of *OPG *expression under either basal conditions (slight) or vitamin D_3 _induction (*p *< 0.06). Interestingly, in the presence of vitamin D_3_, CS upregulated *OPG *expression to a level similar to the one obtained upon treatment with both drugs.

Data from the protein level were almost identical to those from the *OPG *expression, but CS significantly increased *OPG *under both basal and vitamin D_3 _conditions (Figure 2a). GS had no true effect on OPG protein either alone or in combination with vitamin D_3_. The significantly increased levels of OPG with CS and GS incubated together appeared to result from the effect of the CS.

The *RANKL *expression level (Figure 2b) was significantly decreased with CS and with the combination of the two drugs. This decrease again appeared to be the result of the CS effect, as GS upregulated the expression level of *RANKL *at the highest concentration. Vitamin D_3 _drastically upregulated *RANKL *expression. Under this condition, CS alone and in combination with GS tended to downregulate the *RANKL *level. As for OPG, similar values were obtained for CS alone or combined with GS, again suggesting that the effect is related to the CS.

Although we used a specific EIA to determine the total protein level of the RANKL, either in the culture medium or in the cell lysate, the values obtained were at the limit of detection. This is not surprising as, in order to be able to detect quantifiable amounts of RANKL in the culture medium with these human cells and with the available detection EIA, the cells have to be treated with factors such as pro-inflammatory cytokines[26].

The *OPG/RANKL *ratio therefore was determined only from the expression of these factors. However, as the protein levels of OPG correspond to its expression levels, one would expect the ratio calculated with the protein to be similar. Data showed (Figure 2c) that, under basal conditions, the expression ratio of *OPG/RANKL *was significantly increased when cells were incubated with CS alone and in combination with GS. GS alone tended to diminish the ratio in a dose-dependent manner. Vitamin D_3 _significantly decreased the expression ratio of *OPG/RANKL*. Under this treatment, GS diminished the ratio, and a statistically significant decrease was found at the highest concentration.

### Resorption activity

**Figure 3:**
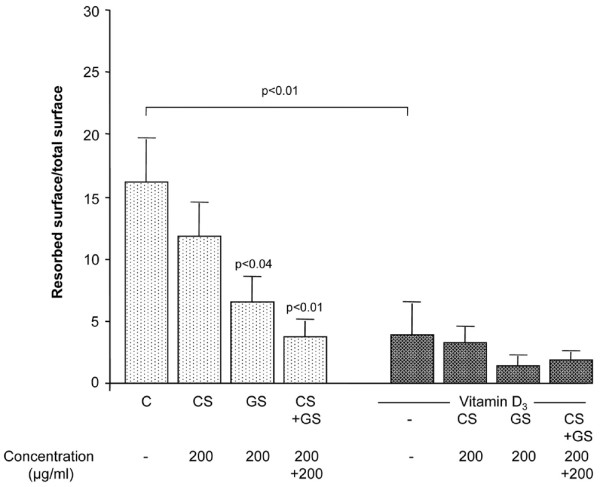
Pro-resorptive activity of human osteoarthritis subchondral bone osteoblasts. Resorption activity of osteoblasts co-incubated with differentiated peripheral blood mononuclear cells in the presence of macrophage colony-stimulating factor and in the absence or presence of chondroitin sulfate (CS) (200 μg/mL), glucosamine sulfate (GS) (200 μg/mL), or both (200 μg/mL each) in the absence or presence of vitamin D_3 _at 50 nM. Data are in the absence or presence of vitamin D_3 _from nine or five independent experiments, respectively. They are expressed as the mean resorbed surface per total surface upon treatment with the factors. Statistical significance was assessed by paired Student *t *test versus autologous control. Underlined *p *value indicates the statistical difference between control (C, basal conditions) and vitamin D_3_-treated specimens.

In our sample, the percentage of resorption in the non-treated specimens was 16.2% ± 3.4% (*n *= 9). Data as illustrated in Figure 3 showed a decrease in the resorption activity when each compound, CS and GS (*p *< 0.04), was incubated alone. The resorption activity decrease became maximal when CS and GS were combined (*p *< 0.01). As expected, vitamin D_3 _significantly reduced this process[11]. Under vitamin D_3_, although there was a tendency to further reduce the resorption activity in the presence of GS alone or CS and GS together, statistical significance was not reached.

The possibility that CS or GS acts directly on the osteoclast formation process was also examined. Experiments were performed as above in which only the PBMCs were inoculated in the well, TRAP staining performed, and the level of multinucleated cells determined. Data showed (*n *= 2) that CS or GS or the two combined do not affect the PBMC differentiation process: levels of 20%, 21%, 19%, and 18% were recorded for control (untreated), CS, GS, and CS and GS, respectively. Similar data were obtained when the PBMCs were co-cultured with osteoblasts (*n *= 4); compared with the untreated control specimens, which were given the value of 100%, CS level was 105% ± 39%, GS 80% ± 34%, and CS + GS 101% ± 43%. Finally, as it has been shown in the literature that some osteoblast lineages produce M-CSF, we investigated whether CS and GS act on the osteoblasts to produce this factor. Experiments were carried out as above (co-culture of PBMCs and osteoblasts) in the presence or absence of M-CSF, and the resorption activity as well as the TRAP intensity were determined. As expected, M-CSF (*n *= 4) induced resorption (22.0% ± 6.0%) and in the absence of M-CSF (*n *= 3) the resorption activity was at a much lower level (2.1% ± 0.1%). Moreover, in the control (untreated) specimens and in the absence of M-CSF, the TRAP staining level was reduced by 25% compared with the level in the presence of M-CSF. However, although the resorbed activity was very low without M-CSF, CS, GS, and the two together appeared to give a pattern similar to that with the presence of M-CSF, in which CS or GS reduced the resorptive activity, and the combination of CS and GS showed almost no resorption.

## Discussion

Bone turnover is the result of a tightly balanced and coordinated action of bone-resorbing and bone-forming elements. These elements are regulated by various factors, including cytokines, growth factors, and extracellular matrix components. The latter include proteoglycans and GAGs such as CS, heparan sulfate, and dermatan sulfate, which are either associated with the cell membrane or stored in the extracellular matrix.

The recent identification of RANKL, its cognate receptor RANK, and its decoy receptor OPG has opened a new molecular field perspective on osteoclast/osteoblast biology and bone homeostasis. We previously demonstrated that OA subchondral bone osteoblasts can be discriminated into two subgroups and that both *OPG *and *RANKL *expression levels, and consequently the expression ratio of *OPG/RANKL*, differ according to the metabolic state of human OA subchondral bone osteoblasts: *OPG/RANKL *is decreased in L- and increased in H-OA osteoblasts[11]. Moreover, the previous study[11] and that of Couchourel and colleagues[14] showed that the metabolic state of the L-OA osteoblasts promotes bone resorption whereas that of the H-OA favors reduced resorption. Indeed, in L-OA osteoblasts, a higher level of differentiated osteoclasts and a thinner subchondral bone mass were observed compared with the H-OA osteoblasts[11] and H-OA osteoblasts demonstrated a higher level of bone deposition[14]. As we wanted to investigate the effects of CS and GS on the remodelling process, we selected the L-OA subchondral bone osteoblast subpopulation. Of note, the osteoblasts from human subchondral bone have already been shown to be mature differentiated cells and, as reported in the literature[12,13,16-21], they express and produce the bone-specific marker alkaline phosphatase, and the level of osteocalcin was drastically increased following vitamin D_3 _treatment.

GS as well as CS have both been tested as therapeutic agents in the treatment of OA) [27-29] Although their clinical efficacy has been demonstrated, the mechanisms by which they mediate their action are not yet fully known. We first examined the effect of CS and GS on alkaline phosphatase and osteocalcin in order to evaluate whether these agents could alter the level of bone markers of terminally differentiated osteoblasts. Upon treatment with CS and GS, both bone phenotypic cell markers were unaffected under basal conditions. Furthermore, cells were also treated with vitamin D_3_, which is known to stimulate both osteocalcin and alkaline phosphatase. Vitamin D_3_, as expected, enhanced the level of these two bone biomarkers[16,17], but CS and GS still did not further affect them. This strongly suggested that both compounds were without effect on the cell integrity.

On the OPG and RANKL system, our data revealed that CS can modulate the expression of these molecules by increasing *OPG *and decreasing the gene expression level of *RANKL*, thereby increasing the mRNA ratio of *OPG/RANK*L. The effect of CS on *OPG *mRNA versus protein could be explained by the following. OPG contains a heparin-binding domain to which some GAGs were demonstrated to bind[30]. Therefore, one can speculate that extracellular CS may bind the OPG heparin domain, thereby enhancing OPG bioavailability by preventing it from being degraded. Furthermore, extracellular OPG was recently shown to modulate the half-life of membranous RANKL by enhancing its degradation through an internalization process[31].

Glucosamine is known to participate in the increased production of GAG and proteoglycans such as aggrecan in cells[32]. Therefore, in following the aforementioned line of thought, we expected to encounter a similar effect with the GS as with the CS. However, the expression ratio of *OPG/RANKL *obtained when cells were treated with GS was not increased. This could be explained by the unlikelihood of modulation of extracellular OPG through a direct interaction with GS, as the affinity of GS, being a monosaccharide, toward OPG heparin-binding domain is expected to be very weak. Indeed, it has been demonstrated that even a tetrasaccharide has a very low binding affinity toward OPG heparin domain compared with a molecule containing more saccharides (that is, hexasaccharide, octosaccharide, and decasaccharide) [30].

Nonetheless, it should not be excluded that CS and GS may also act indirectly through the production of other factors that in turn modulate OPG/RANKL and/or resorption activity. In this context, explorative experiments were carried out in which we looked at whether CS and GS affected the osteoclast differentiation levels and/or the production of M-CSF. Data showed no such effects with these drugs.

Recent studies reported that RANKL-independent mechanisms could also be involved in orientating the bone remodelling toward either a bone resorption or a bone formation process. Thus, one can postulate that such factors could have been modulated by CS and/or GS, thereby indirectly affecting bone resorption activity. Such CS- and/or GS-independent effects could explain our findings in which, although an increase in the OPG/RANKL ratio is found upon treatment with CS, it appears insufficient to significantly reduce bone resorption. The additive effect of both compounds at inhibiting bone resorptive activity could then be explained by the sum of the effect of CS on the OPG and RANKL and the effect of one or both of these compounds on RANKL-independent mechanisms on osteoclastogenesis.

Among the RANKL-independent mechanisms, the following provide interesting hypotheses. Small proteoglycans such as decorin and biglycan are composed of CS chains[8]. These small proteoglycans are able to sequester the transforming growth factor-beta (TGF-β) released by the OA osteoblasts [12], thereby inhibiting the direct stimulatory effect of TGF-β on osteoclast formation) [33-35]. Moreover, GS also demonstrated on an articular cell, the chondrocyte, a RANKL-independent effect on osteoclastogenesis by inhibiting the expression of genes needed for the completion of the osteoclastogenesis process. Indeed, it was demonstrated that interleukin-1-beta (IL-1β) mediates through a RANKL-independent mechanism the multinucleation and the activation of osteoclasts[36] and that treatment with GS prevents IL-1β effects) [37-39] GS was also shown, on chondrocytes, to directly inhibit the activation of the transcription factor nuclear factor-kappa B[38,39], thus preventing the activation of a gene required for osteoclastogenesis. It should be noted that, in the resorption assay used in the present study, cells are incubated with the factors for as long as 3 weeks; therefore, the effect of the drugs on growth factors, cytokines, and transcription factors could very well prevail. Hence, GS, through RANKL-independent mechanisms, and CS, through RANKL-dependent and -independent mechanisms, may explain the additive reduced resorption upon treatment with these two factors.

Data showed that vitamin D_3 _had no effect on the OPG gene expression and protein levels but markedly increased RANKL and, as a result, significantly inhibited the expression ratio of *OPG/RANKL*. These findings agree with the recent literature showing that vitamin D_3 _acts on osteoblasts, thereby increasing RANKL[40] and decreasing OPG[41,42] However, in our study, even though vitamin D_3 _decreased the OPG/RANKL ratio in favor of osteoclastogenesis, a significant decrease in the resorptive activity was observed. This indicates that the RANKL-induced osteoclast differentiation from the differentiated PBMC/osteoblast co-culture system was significantly inhibited by vitamin D_3_. The inhibition of the resorption activity of OA osteoblasts with vitamin D_3 _could relate to a direct effect of this factor on osteoclasts. Indeed, Itonaga and colleagues[43] showed a marked decrease in the formation of TRAP^+ ^and VNR^+ ^(vitronectine receptor^+^) multinucleated cells from PBMCs when treated with vitamin D_3 _and suggest that this factor inhibits osteoclastogenesis through a direct effect on osteoclast precursors.

There are conflicting reports in the literature on the effects of factors, including vitamin D_3_, on the *OPG *and *RANKL *expression levels on bone cells) [44-46] The absence of a consensus may be linked to the use of different experimental model systems, species (rat, mouse, human, and so on), sources of osteoblasts (trabecular or subchondral bone), culture conditions, and the physiological/pathological states of cells. Most reports are from experiments performed on cells from animals and on trabecular bone. Moreover, Thomas and colleagues[47] reported that vitamin D_3 _regulates the *OPG/RANKL *expression ratio differently, depending on the stage of maturity of osteoblasts. Thus, the different findings in the present study compared with some of those in the literature could be due to the use of human specimens from patients with OA, the fact that the osteoblasts are mature but at a particular stage of the disease, and that osteoblasts are from the subchondral bone.

## Conclusion

Our study provides new and interesting data on the effect of CS and GS on human OA subchondral bone osteoblast metabolism. Our data indicate that these compounds, alone or in combination, do not overly affect OA subchondral bone cells. However, CS demonstrated a direct effect at curbing the production of OPG and RANKL, two major factors involved in the remodelling process, and GS significantly reduced the resorptive activity, resulting, when both CS and GS are combined, in a marked reduced resorptive activity. These findings, in addition to the results of studies exploring the effects of these compounds on the catabolic pathways of OA, provide interesting and insightful information about the mechanisms by which these drugs could exert positive effects on the OA disease process.

## Abbreviations

CS = chondroitin sulfate; C_T _= threshold cycle; DMEM = Dulbecco's modified Eagle's medium; EIA = enzyme immunoassay; FCS = fetal calf serum; GAG = glycosaminoglycan; GAPDH = glyceraldehyde-3-phosphate dehydrogenase; GS = glucosamine sulfate; H-OA = high-osteoarthritis; IL-1β = interleukin-1-beta; L-OA = low-osteoarthritis; M-CSF = macrophage colony-stimulating factor; OA = osteoarthritis; OPG = osteoprotegerin; PBMC = peripheral blood mononuclear cell; PCR = polymerase chain reaction; PGE_2 _= prostaglandin E_2_; RANKL = receptor activator of nuclear factor-kappa B ligand; RT = reverse transcription; TGF-β = transforming growth factor-beta; TRAP = tartrate-resistant acid phosphatase.

## Competing interests

JV and EM are employees of and holders of stocks and options in Bioiberica, S.A. (Barcelona, Spain). JM-P and J-PP have received consultancy fees from Bioiberica, S.A. All other authors declare that they have no competing interests.

## Authors' contributions

JM-P participated in study design, analysis and interpretation of data, manuscript preparation, and statistical analysis. SKT participated in study design, acquisition of data, analysis and interpretation of data, manuscript preparation, and statistical analysis. JV and EM participated in study design. DL participated in acquisition, analysis, and interpretation of data. HF and ML participated in acquisition of data. J-PP participated in analysis and interpretation of data and manuscript preparation. All authors read and approved the final manuscript.
